# Fracture as an Independent Risk Factor of Dementia: A Nationwide Population-Based Cohort Study

**DOI:** 10.1097/MD.0000000000000188

**Published:** 2014-12-05

**Authors:** Chun-Hao Tsai, Chieh-Sen Chuang, Chih-Hung Hung, Cheng-Li Lin, Fung-Chang Sung, Chih-Hsin Tang, Horng-Chang Hsu, Chi-Jung Chung

**Affiliations:** From the Graduate Institute of Clinical Medicine (C-HT, H-CH); Department of Orthopedics, China Medical University Hospital, Taichung (C-HT, C-HH, C-HT, H-CH); Department of Neurology, Changhua Christian Hospital, Changhua (C-SC); Department of Life Sciences, National Chung-Hsing University (C-SC); Management Office for Health Data, China Medical University Hospital (C-LL); Department of Public health, China Medical University (C-LL, F-CS); Department of Pharmacology, School of Medicine (C-HT); Department of Medical Research, China Medical University Hospital (C-JC); and Department of Health Risk Management, China Medical University, Taichung, Taiwan (C-JC).

## Abstract

Dementia is among various diseases affecting the elderly, who is also at a high risk for fractures. This study aimed to evaluate the association between fracture history and sequential risk of dementia in Taiwan.

A retrospective cohort study was designed using the claims data of the entire insured residents covered by Taiwan's universal health insurance from 1998 to 2010. A total of 66,797 patients with fractures and 133,594 control subjects without fractures were matched in terms of age (±5 years), sex, and index year and then recruited. Fractures and dementia were defined in accordance with the International Classification of Diseases, 9th Revision, Clinical Modification. The influence of fractures on the risk of dementia was analyzed using a Cox proportional hazards model.

After a 12-year follow-up period, 2775 and 3991 incident cases of dementia were reported in exposed and unexposed cohorts, respectively. The overall incidence rate of dementia in individuals with fractures was 41% higher than that in individuals without fractures (6.05 vs 4.30 per 1000 person-years) at an adjusted hazard ratio of 1.38 (95% confidence interval 1.32–1.45) after age, sex, urbanization, and individual disorders or comorbidities were adjusted. Considering fracture location, we found that patients with hip fractures were at a slightly high risk for dementia. The occurrence of multiple fractures at a single visit was also significantly associated with an increased risk of dementia.

Fracture history is regarded as an independent risk factor of dementia in individuals aged ≥65 years, particularly those who suffered from multiple fractures and/or fractures located in the hip. Further studies are needed to support an independent role of fracture in dementia considering the clinical information and other comorbidities.

## INTRODUCTION

Dementia is characterized by slow progressive memory loss, impaired cognitive function, and inability to perform personal daily activities. Approximately 35.6 million individuals suffered from dementia worldwide in 2010; the number of affected individuals is expected to increase twice in every 20 years and may reach 65.7 million in 2030 and 115.4 million in 2050^38^; as such, dementia is considered as one of the common reasons that individuals are admitted in long-term care units for. This condition is also reported as one of modern society's greatest public health problems. However, the exact pathogenesis of dementia remains unknown. Previous studies identified possible risk factors, including advanced age, female sex, low educational level, family history, and apolipoprotein E genotype.^61^ Other risk factors, such as cardiovascular diseases, depression, head trauma, and diabetes mellitus, have also been identified.^45^

Studies have shown that individuals who suffer from dementia or cognitive impairment are at a substantially higher risk for sustaining a hip fracture than those who are cognitively intact.^13,16,21,39,49^ In Australia, the prevalence rate of dementia in fall-related incidents leading to hip fracture hospitalizations is approximately 24% to 29%, and patients suffering from hip fracture and dementia exhibit a greater mortality rate than those without dementia.^51^ Other studies have reported that delirium, which is one of the main predictors of dementia, may occur following hip fracture surgery.^14,25,35,36^ However, studies have yet to determine whether or not a previous fracture increases the risk of dementia. Therefore, we conducted this nationwide population-based retrospective cohort study by using the database of a universal insurance program to evaluate the association between fracture history and the risk of dementia.

## METHODS

### Study Design and Participants

The Taiwan National Health Insurance (NHI) program was established in 1995; in this system, 13 insurance programs are consolidated in 1 universal system of health care for all residents. The NHI program exhibited a coverage rate of approximately 99% among 23.74 million Taiwan residents and provided contracts to 97% of hospitals and clinics in 2009 (http://nhird.nhri.org.tw/en/index.htm).

Patient data from the NHI research database (NHIRD) were scrambled to generate relevant information. We performed this retrospective cohort study by using a randomly selected population of 1 million insured subjects. All of the patients aged ≥20 years and diagnosed with fracture from 1998 to 2010 were identified as the fracture cohort. The remaining patients without fracture history during the same period were grouped as the nonfracture cohort. However, patients who revealed a history of dementia at recruitment were excluded from both cohorts. The nonfracture cohort was frequency-matched at a ratio of 1:2 for sex, age (every 5 years), and index-year with the fracture cohort. To calculate all of the incident cases of dementia, we set the follow-up period from 1998 until withdrawal or until December 31, 2010, whichever came first. We then analyzed whether or not fracture is associated with an increased risk of developing dementia. The study was approved by the Institutional Review Board of China Medical University (CMU-REC-101-012).

### Definition of Variables

The explored variables included age, sex, urbanization, and comorbidity. Urbanization was categorized into 4 levels based on the population density of a residential area, in which level 1 was considered as the most urbanized and level 4 was considered as the least urbanized. The International Classification of Diseases, Revision 9, Clinical Modification (ICD-9-CM) codes was used to define the diagnoses. The fracture cohort consisted of patients diagnosed with fracture (ICD-9-CM 800–829) based on primary discharge diagnosis. The nonfracture cohort consisted of other insured individuals without fractures. We identified new dementia events (ICD-9-CM 290, 294.1, 331.0) from outpatient and inpatient medical records and confirmed these events with at least 3 medical visits to increase the validity of the diagnoses. Physicians diagnosed dementia based on medical history, psychological tests, physical and neurological exams, blood tests, and brain imaging to rule out other diseases with dementia-like symptoms. The diagnostic criteria used in Taiwan were in accordance with the Diagnostic and Statistical Manual of Mental Disorders, Fourth Edition.^59^ Patients with at least 1 service claim from 1998 to 2010 for either outpatient or inpatient care at recruitment were evaluated for comorbidities, such as diabetes (ICD-9-CM 250), hypertension (ICD-9-CM 401–405), stroke (ICD-9-CM 430–438), coronary artery disease (ICD-9-CM 410–414), head injury (ICD-9-CM 850–854, 959.01), depression (ICD-9-CM 296.2, 296.3, 300.4, and 311), and cognitive impairment (ICD-9 331.83, 438.0, 310.8, and 294.9). To evaluate the severity of fractures, we also analyzed the numbers and locations of fractures, such as vertebrae (ICD-9-CM 806.20–806.9), upper limbs (ICD-9-CM 810, 812–813), hip (ICD-9-CM 820), and thigh/leg/ankle (ICD-9-CM 821, 823–825). We defined multiple fractures as ≥2 fractures in a single visit. A secondary fracture was defined as the recurrence of fracture after more than a year.

### Statistical Analysis

The distribution of categorical variables and the proportions of comorbidities were compared and examined using the *χ*^2^ test between fracture and nonfracture cohorts. The incidence rate ratio (IRR) and 95% confidence interval (CI) of the risk of dementia were evaluated using Poisson regression models. Cox proportional hazards models were used to investigate the association between fracture history and the risk of developing dementia over time, after age, sex, urbanization, and comorbidities of diabetes, hypertension, stroke, coronary artery disease, head injury, depression, and cognitive impairment were adjusted. We further analyzed whether or not the risk of dementia varies when these factors were stratified with the duration of the follow-up period after fractures were diagnosed. For individuals aged ≥65 years, the risk of dementia stratified with varied locations and numbers of fractures were investigated. We compared the effects of fractures on the risk of Alzheimer disease (ICD-9-CM 331.0) and dementia. Statistical analyses were performed using the SAS statistical package (version 9.2 for Windows; SAS Institute Inc, Cary, NC). A 2-tailed *P* < 0.05 was considered significant.

## RESULTS

### Demographics and Sample Characteristics

A total of 66,797 and 133,594 individuals were included in the fracture cohort and the nonfracture cohort, respectively (Table 1). Both cohorts were similar in terms of age and sex distribution with mean age values of 51.4 years in the fracture cohort and 50.9 years in the nonfracture cohort. The prevalence of comorbidities including all of the individual disorders was higher in the fracture cohort than in the nonfracture cohort (Table 1).

**TABLE 1:**
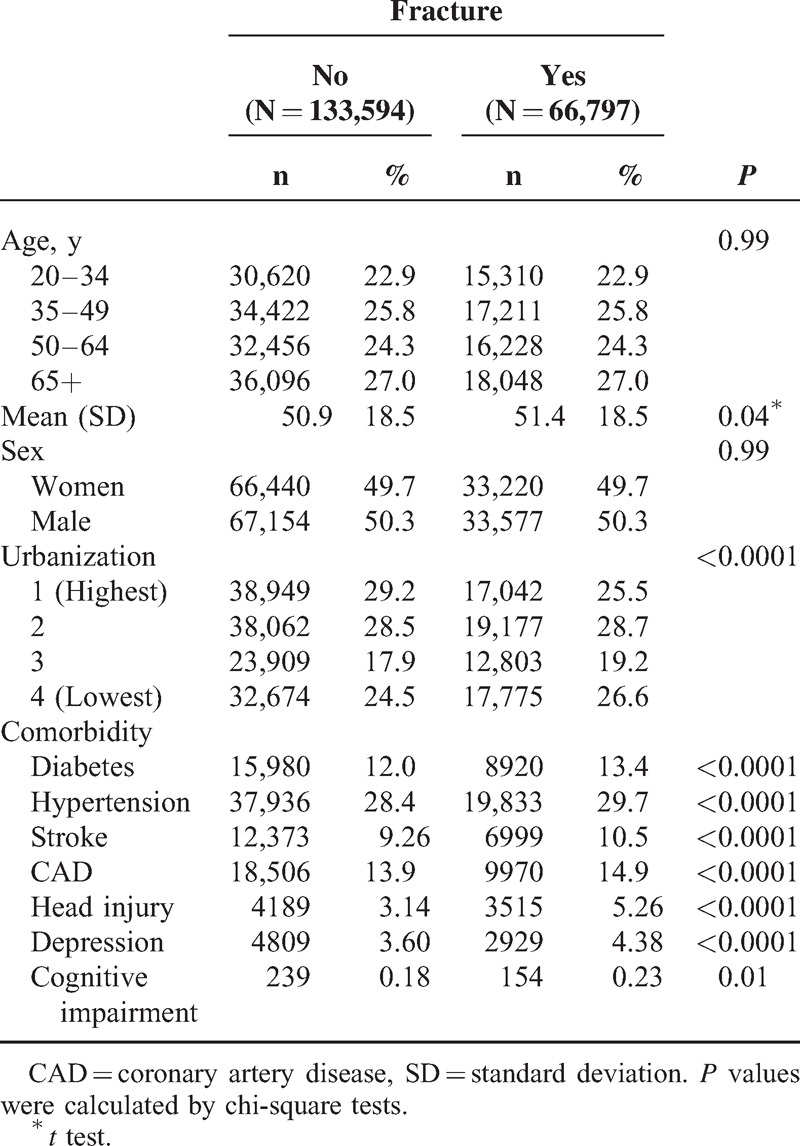
Comparison of Demographics and Comorbidity Between Fracture Patients and Controls

### Association Between Fractures and Incidence of Dementia

**FIGURE 1:**
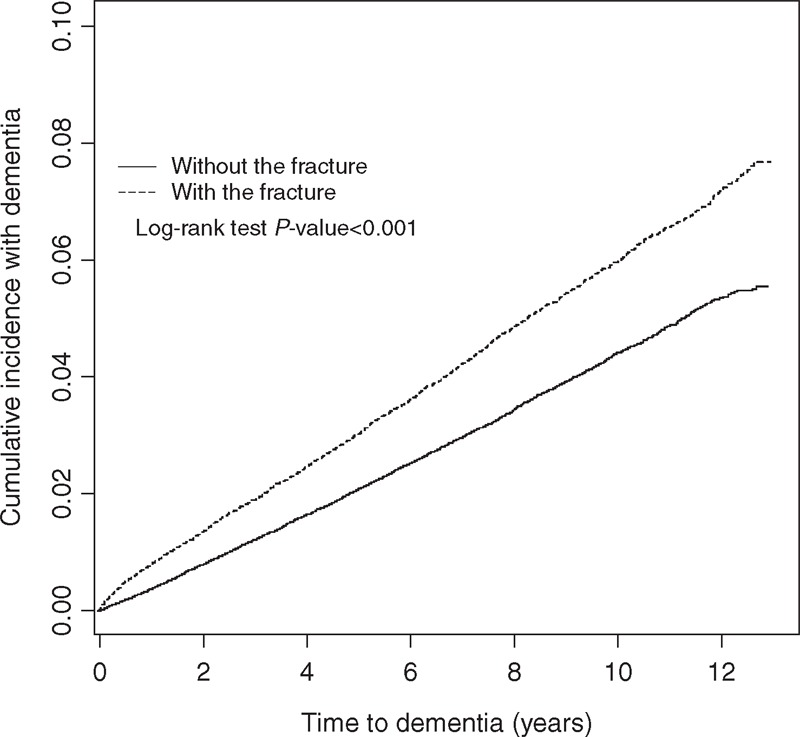
Cumulative incidence of dementia compared between with (dashed line) and without (solid line) fracture.

After the 12-year follow-up period, a total of 2775 and 3991 incident cases of dementia were reported in exposed and unexposed cohorts, respectively. Mean durations during the index year of fracture and dementia incidence were 6.86 (SD = 3.38) and 6.95 (SD = 3.36) in exposed and unexposed cohorts, respectively. The incidence rate and adjusted hazard ratio (AHR) between the exposed cohort and the unexposed cohort are shown in Table 2. The overall incidence rate of dementia was 41% higher in the exposed cohort than in the unexposed cohort (crude rate of 6.05 vs 4.30 per 1000 person-years, respectively) with a crude HR of 1.41 (95% CI 1.37–1.45). After adjusting for age, sex, urbanization, and individual disorders or comorbidities, we found that AHR was 1.39 (95% CI 1.32–1.45). Approximately 1.3- to 1.5-fold risk of dementia was shown after the factors were stratified with the follow-up duration. We also observed a similar positive association after stratification with sex, age, and urbanization was performed. The incidence rates of dementia were the highest in the elderly (age ≥65 years) in both cohorts (16.3 vs 23.1 per 1000 person-years). The IRR of dementia for the youngest age group (20–34 years) was also high (IRR = 4.95, 95% CI 4.56–5.39) with an AHR of 4.48 (95% CI 1.96–10.2). A significantly increased risk of dementia was similarly observed regardless of the presence of comorbidities. The results of the log-rank test and cumulative incidence curve of dementia showed that the patients with fractures had a significantly higher incidence of dementia than the subjects in the comparison cohort (log-rank *P* < 0.001) (Figure 1).

**TABLE 2:**
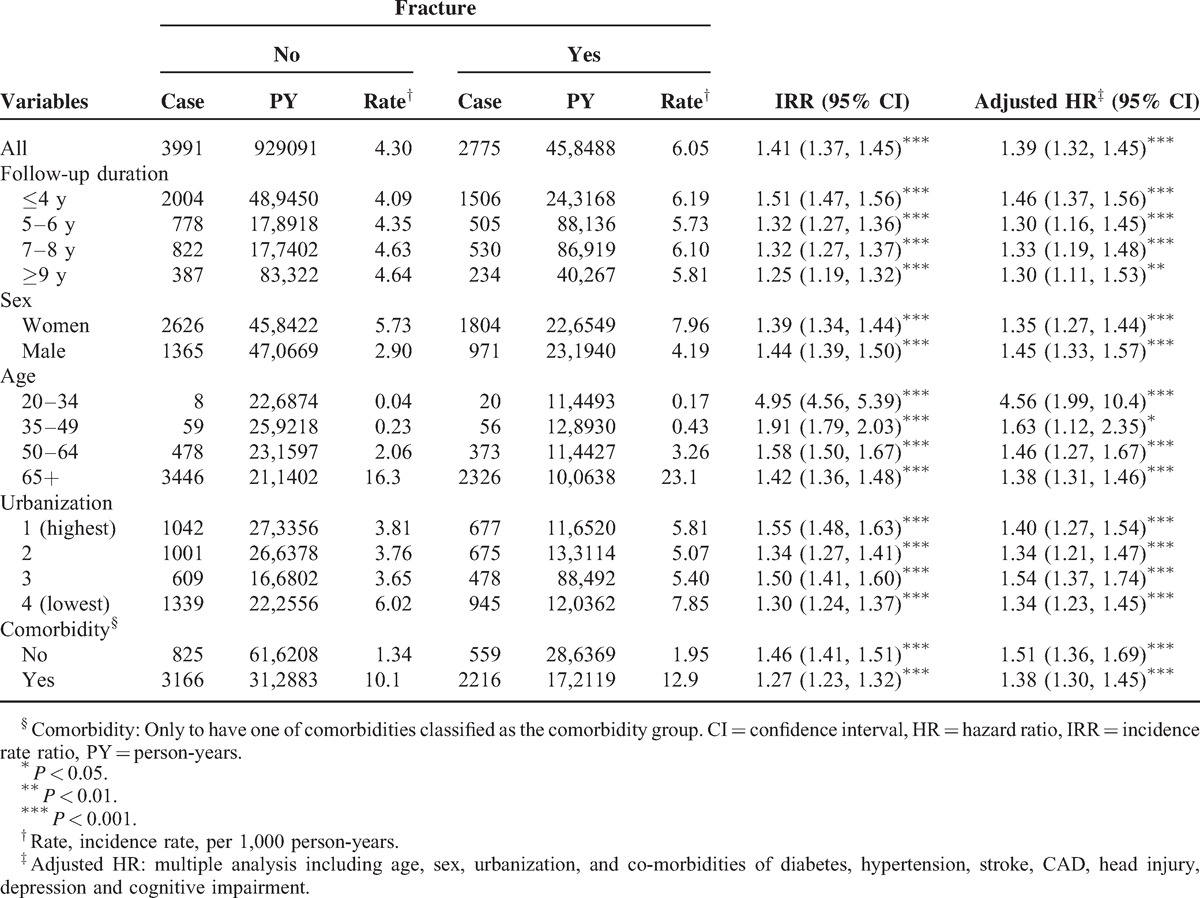
Incidence and Adjusted HR of Dementia Stratified By Sex, Age, and Comorbidity Compared Between Patients With Fracture and Without Fracture

The incidence rate of dementia was higher in patients with comorbidities than in patients without comorbidities. The positive associations of fracture and dementia ranged from 1.3- to 1.5-fold risk after other potential risk factors were adjusted (data not shown). Table 3 shows the incidence rates and AHR of dementia according to the number and location of fractures in individuals aged ≥65 years. The patients with hip fractures exhibited a 60% higher risk of dementia (AHR 1.60, 95% CI 1.43–1.79), those with fracture in the vertebrae revealed a 47% higher risk (AHR 1.47, 95% CI 1.11–1.94), those with thigh/leg/ankle fracture presented a 35% higher risk (AHR 1.35, 95% CI 1.18–1.55), and those with an upper limb fracture displayed a 29% higher risk of dementia (AHR 1.29, 95% CI 1.17–1.43) compared with the unexposed cohort. It suggested that those with hip fractures had slightly high risk of dementia. Patients with multiple fractures also exhibited a significantly increased risk of dementia. We further distinguished the incidence of Alzheimer disease from dementia and analyzed the data in Table 4. Similar risks of positive associations were observed in Alzheimer disease and dementia (AHR 1.29, 95% CI 1.04–1.61 and AHR 1.39, 95% CI 1.32–1.46, respectively).

**TABLE 3:**
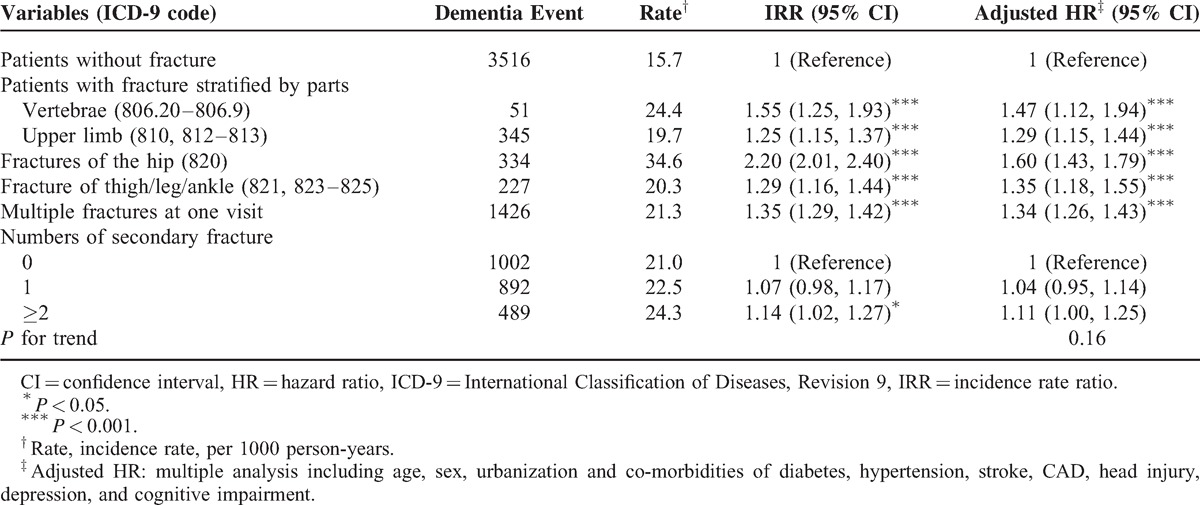
Incidence and Adjusted HR of Dementia Stratified by Different Parts and Numbers of Fracture in Patients aged ≥65 years

**TABLE 4:**
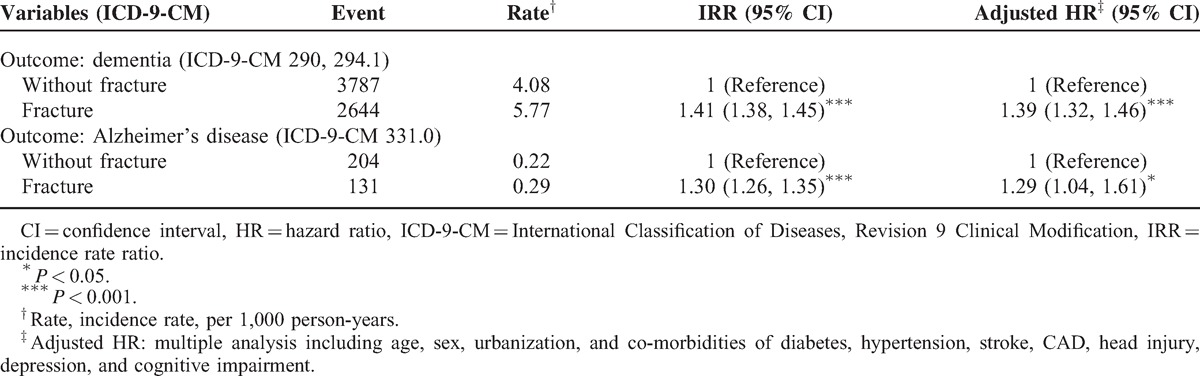
Comparisons of HR Between Patients With and Without Fracture for Different Outcomes (Dementia and Alzheimer's Disease)

## DISCUSSION

The current study demonstrated that fracture history could lead to an increased risk of dementia, particularly in individuals aged ≥65 years and suffered from multiple fractures and/or fracture/s in the hip.

Dementia and fractures are 2 adverse conditions commonly affecting the elderly; both conditions are associated with the same frailty-related risk factors, such as old age, vitamin D deficiency, presence of apolipoprotein E4, and lifestyle.^13,32^ Patients with low bone mineral density are at a high risk for fractures, and this condition is associated with low hypothalamic volume in the early onset of Alzheimer disease; this result suggests that the central mechanisms of bone remodeling may be disrupted by neurodegeneration.^31^

The cause of dementia remains uncertain and may include multiple factors. To our knowledge, no histological or animal study has shown that fracture can directly cause sequential dementia. The possible biological mechanism may be associated with the formation of excess superoxide radicals during fracture healing. In the first few weeks after fracture, oxidative stress likely occurs, as excess superoxide radicals are produced to remove debris.^50^ The overproduction of reactive oxygen species induces endothelial dysfunction, thereby leading to vascular dementia and causing increased amounts of amyloid-beta peptides in patients with Alzheimer disease.^5^ After fracture, consequences, such as decline in physical function, chronic pain, and prolonged inflammatory cytokine secretion during fracture repair, may contribute to dementia.

The quality of life after an individual suffers from fracture has an important influence on the development of dementia. For instance, a decline in physical function and changes in appearance may contribute to a loss of social engagement and physical exercise; as a result, the quality of life of patients is impaired. Fracture has also been considered as an independent influencing factor of the development of functional decline in the elderly regardless of prefracture health conditions.^47^ Chronic pain after an individual suffers from fracture also contributes to cognitive decline in later life. After suffering from fracture, 7% of patients likely develop complex regional pain syndrome, and none of these patients are relieved from this symptom after 1 year.^3^ Chronic pain also affects approximately 48.4% of patients after these individuals suffer from pelvic fracture.^40^ The interaction of pain and psychiatric depression is a vicious cycle, that is, pain causes depression and depression causes pain.^7,11,18,28,34^ Furthermore, chronic pain can be considered as a brain disease, in which alterations in neural networks affect multiple aspects of brain function, structure, and chemistry; such changes also induce behavioral and psychiatric symptoms.^6,55^ Pain also affects cognitive function and elicits changes in the central nervous system, specifically on emotional processing.

A fracture may increase the rate of degeneration via inflammation that contributes to cognitive decline. Neuroendocrine responses mediate macrophage function after trauma.^33^ For example, interleukin-6 (IL-6) regulates the differentiation of osteoblasts and osteoclasts; IL-6 also promotes angiogenesis by stimulating the release of vascular endothelial growth factors.^62^ IL-6 concentrations remain high for several months after fracture^41,44^; such high concentrations may cause bone remodeling and bone loss.^43^ IL-6 has also been considered as a key factor in inflammation in the pathophysiology of depressive symptoms after individuals suffer from hip fracture.^37^ High levels of IL-6 have also been reported in elderly patients suffering from hip fracture and impaired mental status.^4^ Furthermore, increased IL-6 levels are associated with an increased risk of Alzheimer disease.^30^ In addition to these factors, inflammation and increased peripheral cytokine levels are associated with depression-like symptoms and neuropsychological disturbances in humans.^9,63^

The present study found a higher risk of dementia in younger individuals or males with fractures than in other patients. Patients with low or major limb fractures also exhibited an increased risk of dementia compared with individuals suffering from upper limb or spine fractures. Various mechanisms, chronic pain, decreased physical activities, and prolonged inflammatory conditions after patients suffered from fracture may partially explain the differences in the risk of dementia in terms of sex, fracture site, and age. For example, young subjects who experience fracture may suffer from chronic pain and system inflammation for a long period; therefore, these individuals may exhibit an early deterioration of physical status. Alzheimer disease may also exhibit an early onset, in which symptoms are manifested before patients reach 65 years of age. However, only a few population-based studies on the epidemiology of young-onset dementia have been reported. For instance, Harvey et al^17^ estimated that the prevalence of dementia at the onset age of 30 and 65 years is 54 cases per 100,000 individuals (95% CI 45–64); by comparison, the prevalence rate of dementia at the onset age of 30 to 44 years is 12.1 cases per 100,000 individuals. Using a 2-step postal survey with a population of 2,966,000 individuals, Ikejima et al^40^ found a prevalence of 42.3 cases per 100,000 individuals (95% CI 39–45) in the Ibaraki prefecture in Japan between the ages of 18 and 65 years. In the previous study, the most frequent cause of early-onset dementia is vascular dementia (42.5%).^19^ Kelley et al^23^ also evaluated 235 patients with progressive cognitive decline between the ages of 17 and 45 years; the result showed that patients with an onset age of <35 years mainly manifest metabolic causes with inborn errors. Neurodegenerative etiologies are also common when individuals reach 35 years of age. Furthermore, mutations in the genes encoding amyloid precursor protein, presenilin 1, and presenilin 2 are responsible for early-onset autosomal-dominant Alzheimer disease.^15^ One of the functions of amyloid precursor protein is related to the suppression of osteoblastogenesis and bone formation,^60^ implicating amyloid precursor protein as a risk factor of osteoporosis and bone fracture. This finding could partially explain the association between fracture and young onset of dementia. However, the incident cases of dementia affecting 20-year to 34-year age group were small in our present analysis; as such, a wide range of CI was obtained. Hence, further studies should be conducted to investigate the association between fracture and dementia in young populations. In women, the low risk of dementia can be explained by the protective effect of estrogen, which elicits an anti-inflammatory effect that decreases the damage caused by cytokine secretion; this hormone also functions against neurodegeneration.^20,29,42,46,48,53,57^ In addition, surgery for a fracture induces sequential inflammatory processes.^24,54,58^ Further studies should also be conducted to determine whether or not other aging processes, physical conditions, and inflammation mediate fractures and cognitive decline.

Multiple factors can cause dementia after an individual suffers from fracture. Each factor may also exhibit a different effect with time. Interventions to prevent dementia in later life should be multidisciplinary and comprise medical, social, physical, and psychological strategies. For example, antioxidants, as nutrient supplements, help prevent dementia after an individual suffers from fracture.^56^ Vitamin E also produces beneficial effects on new bone formation by reducing lipid peroxidation during early fracture healing^26,52^; foods rich in vitamin E also reduce the long-term risk of dementia.^10^ Furthermore, clinical applications, including early successful stabilization, nutrient supplementation, postoperative rehabilitation, and regular exercise,^12^ help reduce the risk of dementia. Physical activity also reduces the risk of developing dementia, and a higher level of midlife fitness is possibly associated with a low risk of dementia in later life. Moreover, physical activity or exercise is considered as an important protective factor reducing the risk of dementia.^1,2,8,27^ In a previous study, the association among cognitive impairment and exercise, cognitive activities, and socialization was investigated and the result showed that exercise is associated with a low risk of cognitive impairment in the 10-year follow-up period, but this low risk is not statistically significant in the 5-year follow-up period.^22^

The strengths of this study include the use of population-based data that are highly representative of a general population. However, several factors limit this study. First, the NHIRD does not contain detailed information regarding BMI, smoking habit, alcohol consumption, educational status, patient's living conditions (nursing home vs community), and preinjury physical and mental status, which may be considered as risk factors of dementia. In addition, all of the data in the NHIRD are confidential. Thus, relevant clinical variables, such as bone mineral density, fracture severity, surgical methods, and serum laboratory data were unavailable for analysis. Second, the evidence derived from a retrospective cohort study is generally lower in statistical quality than that from randomized trials because of potential bias related to adjustments for confounding variables. Third, we cannot exclude the possibility of bidirectional relationship between facture and dementia because dementia usually has a long preclinical phase. In addition, we could not recruit individuals with mild dementia or others who did not seek medical care in our present analysis. Individuals with fracture are more likely to consult a doctor than those without fracture, and this difference may cause detection bias because of frequent examinations. These factors may also weaken the true association. If individuals who suffer from fracture were more likely to undergo diagnosis for dementia, these individuals could pose a high risk of dementia in early follow-up periods. However, similar risks of approximately 1.3- to 1.6-fold were observed in our analysis. Nevertheless, the data regarding fracture and dementia diagnoses were reliable.

## CONCLUSIONS

In conclusion, a fracture could be considered as an independent risk factor of dementia. A comprehensive understanding of the pathways and their relative effects on the outcomes of fractures could provide the basis of the development of effective interventions and potentially improve preventative efforts. Further studies are needed to support an independent role of fracture in dementia considering the clinical information and other comorbidities.
