# Fracture as an Independent Risk Factor of Dementia: A Nationwide Population-Based Cohort Study: Erratum

**DOI:** 10.1097/01.md.0000490128.42415.8e

**Published:** 2016-07-29

In the article “Fracture as an Independent Risk Factor of Dementia: A Nationwide Population-Based Cohort Study”,^1^ which appeared in Volume 93, Issue 26 of *Medicine*, the authors’ affiliations were given incompletely in the original article. The article has since been corrected online.
